# Shiga Toxin-Producing *Escherichia coli* in Aquaculture: A Decade (2015–2025)-Long Global Retrospective Outlook

**DOI:** 10.3390/vetsci13080779

**Published:** 2026-08-04

**Authors:** Ayesha Sarwar, Bilal Aslam, Sulaiman F. Aljasir

**Affiliations:** 1 Institute of Microbiology, Government College University Faisalabad, Faisalabad 38000, Pakistan; ayesha.sarwar@gcuf.edu.pk; 2 Department of Veterinary Preventive Medicine, College of Veterinary Medicine, Qassim University, Buraydah 52571, Saudi Arabia

**Keywords:** STEC, ARGs, VAGs, aquaculture, antimicrobial resistance

## Abstract

Shiga toxin-producing *Escherichia coli* (STEC) contamination and proliferation in aquaculture and aquatic systems is worrisome for global food safety, as well as veterinary and public health. Traditionally linked with terrestrial ruminant reservoirs, aquaculture matrices, including farmed finfish, shellfish, culture water, and benthic organisms, are increasingly acknowledged as potential conduits for the dissemination of STEC. Herein, the review documented data concerning the prevalence, genomic composition, and ecological dynamics of STEC across various aquaculture environments across different regions of the globe. As a result, the persistence of virulence-associated genes (VAGs) and antibiotic-resistant genes (ARGs) in STEC within the aquaculture supply chain presents a considerable risk to global food security and public health, highlighting the urgent necessity for comprehensive “One Health” surveillance frameworks aimed at alleviating aquatic biosecurity challenges.

## Simple Summary

Aquaculture environments have emerged as a potential source of Shiga toxin-producing *Escherichia coli* (STEC), posing a severe threat to global biosecurity. Driven by horizontal gene transfer, diverse and antibiotic-resistant non-O157 serotypes are rising alongside persistent, traditional O157:H7 lineages. Researchers utilize advanced phenotypic and molecular assays along with genome sequencing to map these virulence and resistance profiles. The survival of these robust pathogens throughout the supply chain demands the urgent implementation of “One Health” surveillance frameworks, which will be beneficial for global food safety.

## 1. Introduction

The role of aquaculture as a major food-producing sector worldwide is illustrated by its progressively increasing share of global fish production. As forecasts suggest, the world aquaculture output could surpass 104 million tons by 2025 [1]. Aquaculture is useful in providing protein for the increasing human population, but there is a limit to this expansion due to the environmental degradation of ecosystems. However, intensive aquaculture that is based on overcrowding causes aquatic ecosystems to deteriorate, making aquaculture facilities prone to microbial contamination. That is why it becomes very important that the link between environmental sustainability and biosecurity is well taken care of to reduce the risk of enteropathogen, i.e., *E. coli*, contamination in the production of aquatic food–water supply chain [2].

The resilience of *E. coli* in aquaculture settings presents a challenge that has been further compounded by various environmental factors that increase the possibility of bacterial proliferation and lateral gene transfer processes. Changes in hydrological dynamics and warming of aquatic bodies have been found to contribute significantly to the shift in *E. coli* from being merely commensal to an active pathogen that can cause serious tissue destruction and even death in fish populations [3]. This pathogenic activity involves the construction of biofilm and release of toxins that affect the mucosa of the host’s intestines [4]. Pathogen strains differ in terms of their genomic organization, particularly in the presence of virulence determinants such as intimin, hemolysin, and invasions [5]. Thus, the aquaculture ecosystem plays a crucial role in transferring these pathogens to humans through the food web [6].

STEC is a leading zoonotic pathogen with a notable feature of being able to induce disease with a small infectious dose. The appearance of STEC as a major public concern in the aquaculture industry reveals another important area of connection between food safety and environmental safety. While STEC was first isolated from terrestrial livestock animals, this pathogen showed an impressive ability to adapt to life in the aquatic environment by forming a resistant biofilm [7]. The pathological condition caused by STEC depends mainly on the presence of cytotoxic toxins *stx*1 and *stx*2, which result in varying severities of disease, including gastroenteritis and Hemolytic Uremic Syndrome (HUS), which is a major cause of acute renal failure in children and consists of microangiopathic hemolytic anemia, thrombocytopenia, and acute kidney injury [8]. The pathogen was usually linked to contaminated cattle products; however, STEC now causes more diseases via contaminated water and blue foods due to its considerable environmental adaptation. Highly toxic strains of the pathogen have the ability to create resistant biofilms in water bodies, and thus strict control is required for the prevention of HUS in the aquaculture chain [9].

The growing rate of AMR in aquaculture is emerging as a public health issue, mainly due to the misuse of antibiotics both in the prevention and treatment of ailments in intensive fish farming. These fish farms become environmental niches of MDR *E. coli*, which affects the health of teleost fishes and leads to zoonotic transmission of resistance genes through the food–water route [10]. Another important factor affecting the spread of AMR among fishes is climatic warming, as it enhances selective pressure and horizontal gene transfer (HGT) of resistance determinants [11]. One of the most concerning aspects of the matter is the occurrence of ESBL genes like *bla*_CTX-M_, *bla*_TEM_, *bla*_SHV_, and *bla*_OXA-1_, as well as carbapenemase resistance genes such as *bla*_NDM_, *bla*_KPC_, *bla*_OXA-48_, and *bla*_VIM_ [12]. Importantly, carbapenemase makes carbapenems useless in fighting MDR Gram-negative infections [13].

STEC infiltration pathways into aquaculture present a complex biosecurity issue that stems from watershed pollution. Although livestock manure-laden runoff continues to be the most common source of pathogen infection, urban wastewater dumping adds an extra layer of danger to this process, especially in highly populated agricultural areas. Aside from anthropogenic pollution sources, wildlife becomes an additional mobile factor in the distribution of STEC across open waterways, creating new routes of contamination that go beyond the scope of traditional land-based prevention techniques [14]. Poor sanitary practices in rural watersheds only aggravate the situation. In this way, the aquatic ponds become bioenvironmental receptors within a dynamic system of microbial exchange, in which the presence of Shiga toxin-producing strains connects environmental damage directly to foodborne illness [15]. The spatial and temporal dynamics of STEC in aquatic environments are characterized by a complex combination of adaptive mechanisms, which allow STEC to withstand the changing physicochemical conditions. Contrary to the case of commensal types of bacteria, STEC strains often acquire a viable, not culturable (VBNC) state in response to various environmental stresses, such as salt concentration, temperature changes, and exposure to ultraviolet light. One of the key strategies adopted for the survival of STEC in water ecosystems involves the synthesis of extracellular polymeric substance (EPS) for forming biofilms on aquaculture equipment, including pond-lining material and filters [16]. Biofilms protect from oxidation and disinfection with traditional methods. Aquatic sediments may also serve as reservoirs for STEC, which can persist within them until random factors cause them to re-enter the water, such as resuspension during harvesting or rainfall [17].

Despite the known hazards associated with STEC in international food systems, a critical knowledge gap has been noted regarding its ecology in the context of the aquaculture industry. Although livestock farming and horticulture have historically been the focus of research, the temporal and spatial characteristics of STEC in fishery settings have yet to be explored sufficiently [18]. Moreover, it should be noted that the scientific community lacks information on the effects of weather phenomena, from seasonal floods to ocean warming, on the incidence of hyper-virulent STEC serotypes. Such geographical biases can be observed in the lack of genotypic data on LEE-negative strains of STEC in tropical aquaculture centers, which have become leaders in international production volumes. Therefore, present monitoring efforts tend to ignore the One Health perspective on the interaction between sediments and wildlife agents in water ecosystems, raising concerns about ensuring the safety of the emerging blue economy [19].

The current research will bridge this significant data gap in understanding STEC persistence in aquaculture by studying its distribution along the entire continuum of aquatic environments. We will ponder the significance of sediment and biofilm communities as microbial sanctuaries, and investigate the presence of STEC and its associated properties in aquaculture systems and the associated environment, focusing specifically on local environmental stressors [18]. The key aspects under study consist of seasonal microbial variation, the persistence of the main VAGs (*stx*1, *stx*2, *eae*A), and the relevant ARGs associated with them. The results obtained are expected to form the basis for implementing biosecurity practices and protecting the blue economy from both anthropogenic contamination and evolving pathogenic niches [19].

## 2. Materials and Methods

A systematic literature review was conducted to evaluate laboratory-confirmed STEC infections. Comprehensive electronic searches were performed through three major databases, PubMed, Web of Science, and Google Scholar, targeting the publication period from 2015 to 2025. To avoid the effects of publication bias and to get a wider epidemiological picture, beyond the peer-reviewed literature search, an evaluation of the gray literature and public health surveillance data was also done. The entire process of searching the databases, literature screening, and data extraction was accomplished from 19 February to 1 November 2024. Various steps involved in the implementation of the search strategy include identification, screening eligibility, and final article selection. This study was conducted according to the Preferred Reporting Items for Systematic Reviews and Meta-Analyses guidelines. The PRISMA Checklist (File S1) was followed for each section of the meta-analysis (https://www.prisma-statement.org/). This systematic review was registered in the International Prospective Register of Systematic Reviews (PROSPERO), under the identifier CRD420261443874 dated 7 July 2026.

### 2.1. Inclusion Criteria

The search terms used in these databases included Medical Subject Heading (MeSH) terms (https://www.nlm.nih.gov/mesh/meshhome.html, accessed on 19 April 2024). These included “Verotoxigenic”, “Verotoxigenic *Escherichia coli*”, “Verocytotoxin Producing *Escherichia coli*”, “Verotoxin-Producing *Escherichia coli*”, “VTEC”, “Shiga Toxin-Producing *Escherichia coli*”, “STEC”, “Enterohemorrhagic *Escherichia coli*”, and “EHEC”, along with the names of developing countries classified by the United Nations in the World Economic Situation and Prospects 2023 report (https://unctad.org/system/files/official-document/wesp2023_en.pdf, accessed on 1 January 2024). While VTEC and EHEC were valid search terms for finding relevant papers in this review, they are now generally considered obsolete or less precise in the scientific community.

### 2.2. Exclusion Criteria

To maintain a focus on novel epidemiological and observational data, we excluded laboratory-centric studies (focusing on isolation or diagnosis), microbiological characterizations of redundant strain sets, and clinical treatment reviews. After an individual assessment of each record against these predefined exclusion criteria, the final dataset comprised 108 peer-reviewed papers.

### 2.3. Systemic Literature Screening

**Figure 1:**
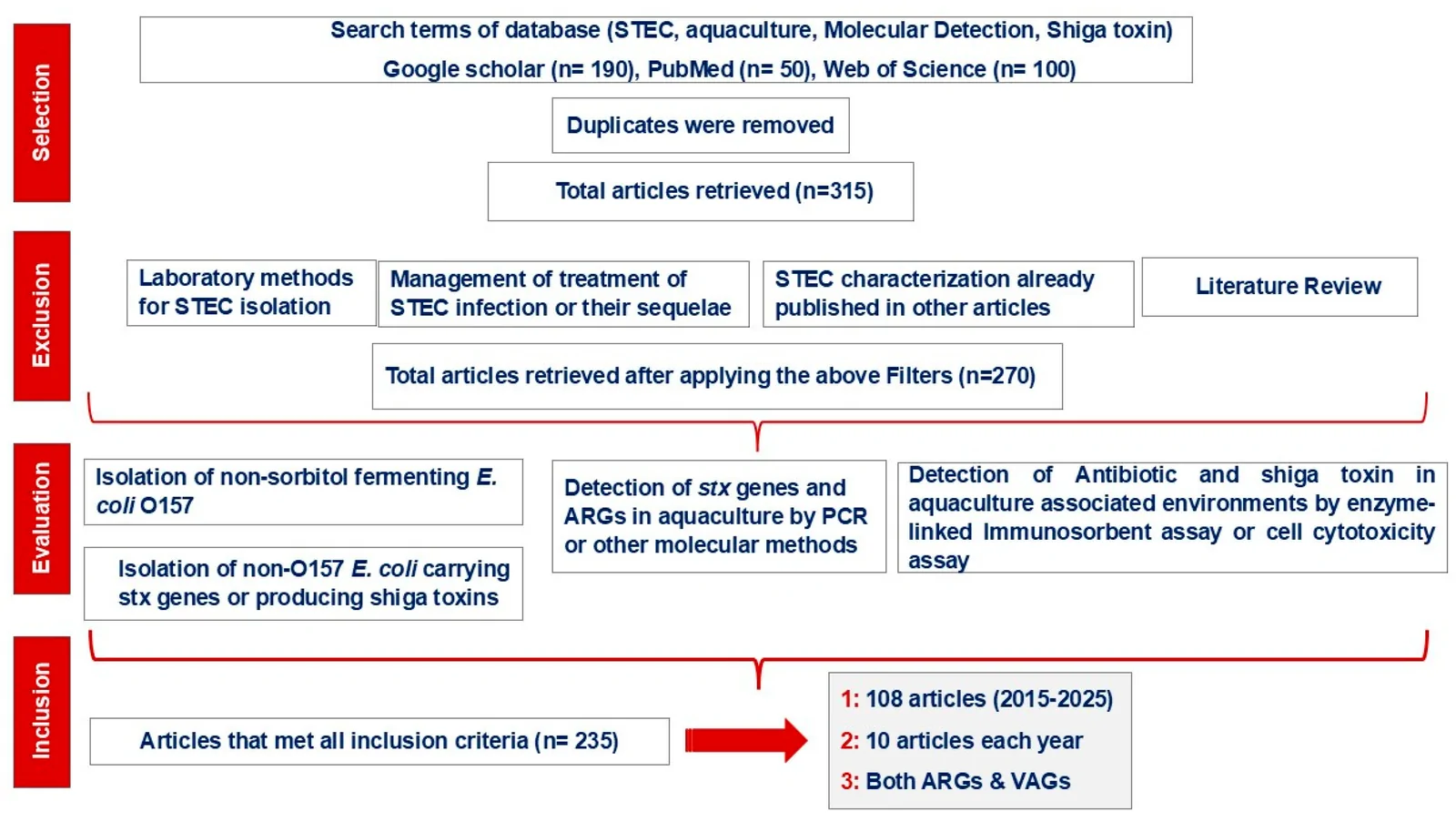
A systemic literature review employed in the form of a flowchart describing the various selection criteria (including different exclusion and inclusion parameters, evaluation approaches and final selection) of STEC distribution from aquaculture and associated environments.

Adopting the widely accepted STEC nomenclature, we performed a systematic screening of 315 non-duplicate, English-language articles. After the initial screening, selected publications were examined to see if they reported the presence of non-sorbitol-fermenting *E. coli* O157 or non-O157 strains that contain the stx genes or produce the Shiga toxin. Studies had to verify STEC in human or animal feces by molecular methods like PCR or by phenotypic tests like ELISA or cell cytotoxicity to be considered eligible for this study. Articles dealing with asymptomatic carriage or urinary tract infections were omitted (Figure 1).

### 2.4. Statistical Analysis

Information from selected papers was combined into a Microsoft Excel 2019 spreadsheet; each entry included study metadata (DOI, year, country) and virulence markers (serotypes, *stx*1/*stx*2, *stx* subtypes, *eae*). Results were then divided by source: human, animal (e.g., ruminants, pigs, and birds), and food (fish farming and its environment). Moreover, data from various continents was subjected to one-way analysis of variance (ANOVA) to compare the means of independent variables, i.e., STEC.

## 3. Results

**Figure 2:**
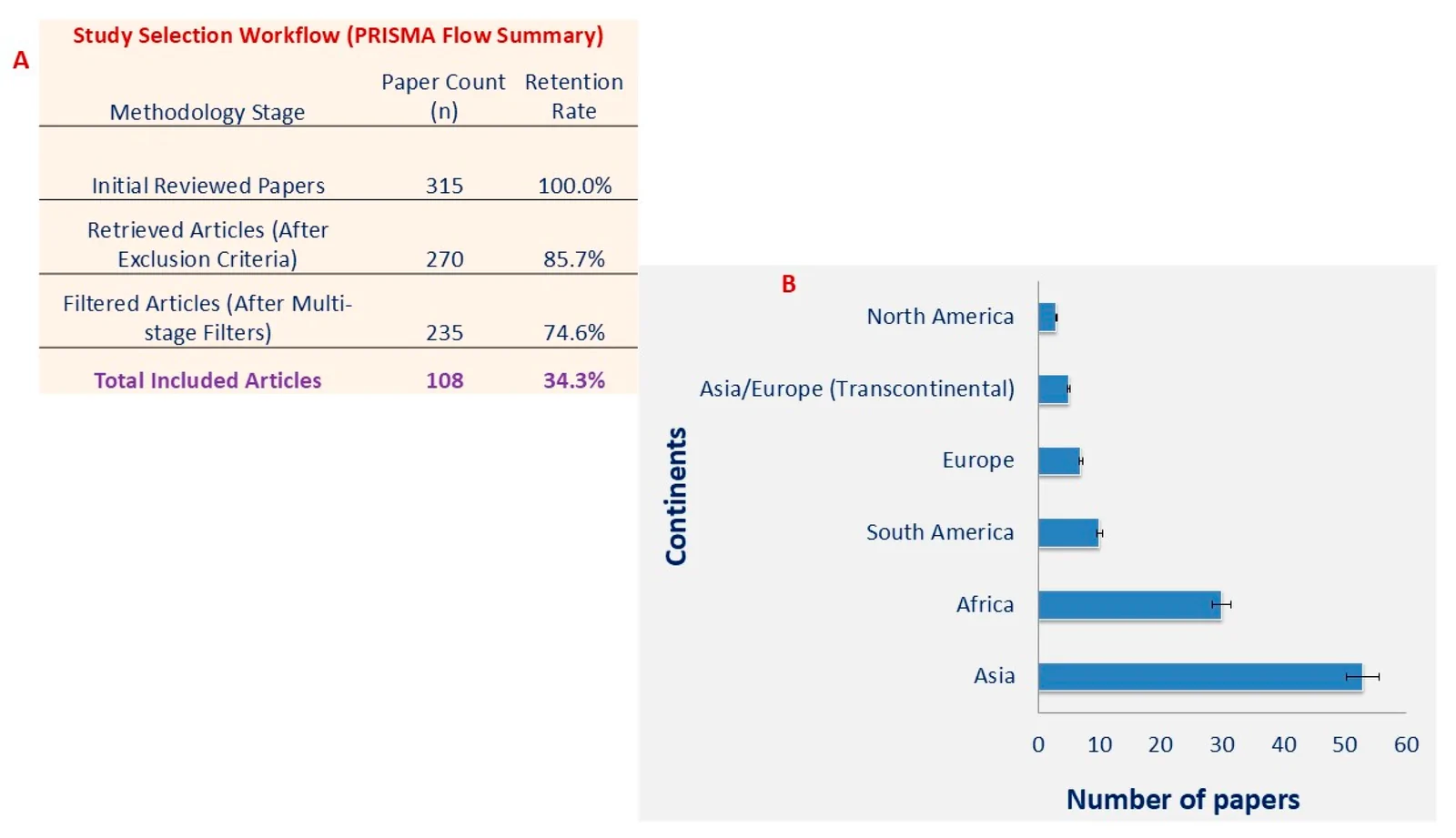
Illustration of the overall distribution of research articles reviewed. (**A**) Summary of total article count (where 315 papers were initially reviewed, which reduced to 270 papers after applying exclusion criteria and 235 after applying multiple filters, leading to a final paper count of 108. (**B**) Geographical representation of the paper count by continent. These blue bars indicate the number of articles reviewed from different continents. The highest number of articles included was from Asia (53) followed by Africa (30), Europe (12), South America (10) and finally the lowest number of articles reviewed was from North America (3).

In this study, after reviewing (*n* = 315) papers and applying different exclusion criteria, (*n* = 270) articles were retrieved, which were evaluated by passing through various filters to get (*n* = 235) articles. After employing final inclusion on the basis of VAG and ARG detection in aquaculture, (*n* = 108) articles were included (Figure 1). Of these 108 articles across six continents, the largest share originated from Asia (53 papers), followed by Africa (30 papers), transcontinental Asia/Europe (5 papers), South America (10 papers), Europe (7 papers), and the lowest number was from North America (3 papers). A total of 42 papers from different countries showed the presence of VAGs, where Shiga toxin genes (*stx*1, *stx*2) and their respective subtypes and the intimin gene (*eae*) were collected from 1419 out of 4082 isolates, while 3055 ARGs were reported among various aquaculture and associated samples (Figure 2).

### 3.1. Distribution of STEC Isolates

#### 3.1.1. Continent/Country-Wise

**Figure 3:**
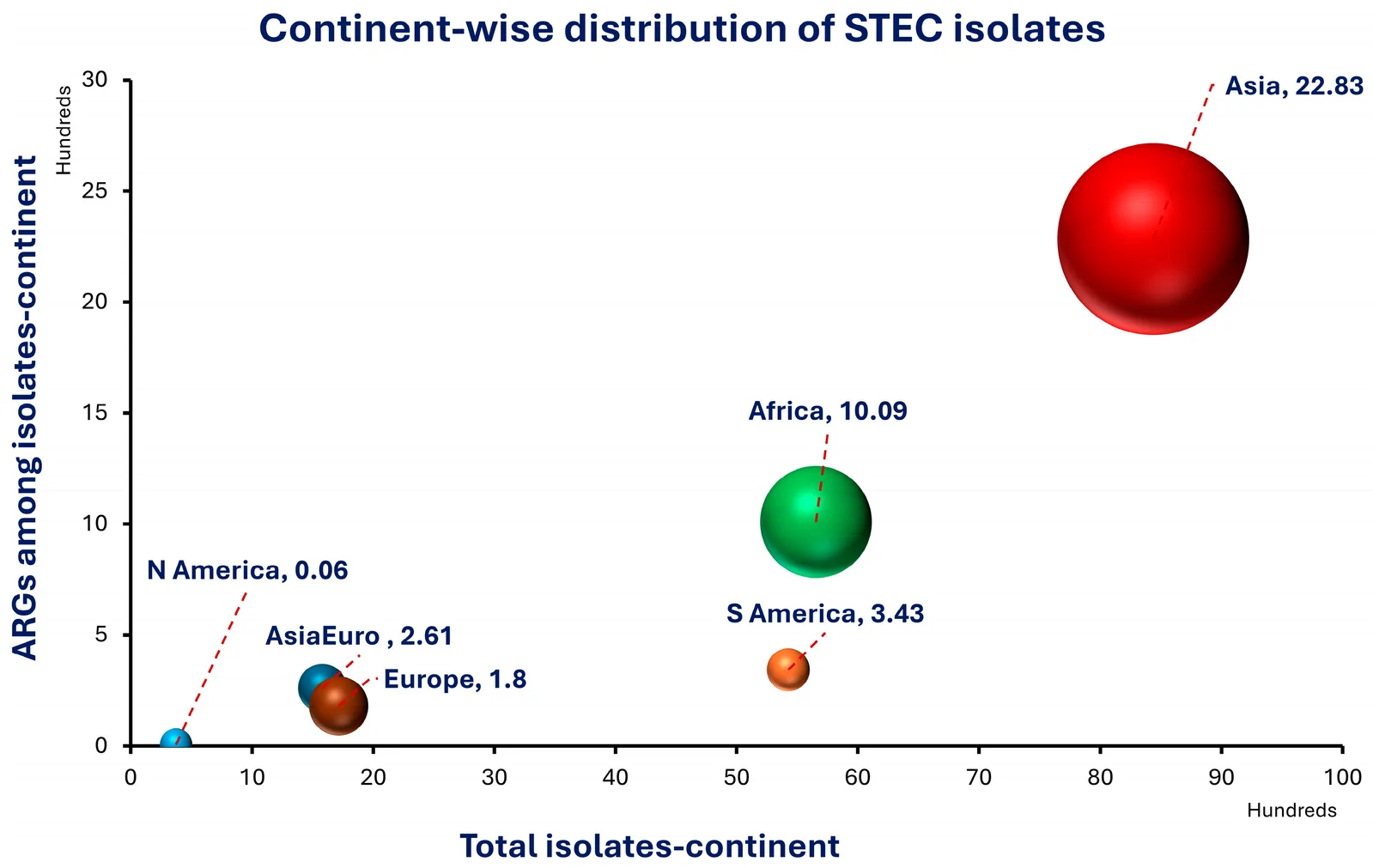
Scattered bubble plot; the size of the bubble represents the number of STEC isolates (×100) in different continents, along with total isolates among continents (x-axis), and occurrence of ARGs among isolates (y-axis).

Among all the 4082 STEC isolates reported worldwide within various aquatic ecosystems (Figure 3), Asia has the largest number of 2283 isolates (approximately 55.92%), owing to sampling in coastal and aquaculture regions of India, along with major contributions from Thailand, Malaysia, Jordan, and Lebanon. Second on the list is the continent of Africa, which accounts for 1009 isolates (approximately 24.33%), mostly from Egypt (the largest contributor within Africa, based on research on fish farms, shrimp, and seafood), with smaller contributions from Zambia, Nigeria, Ethiopia, and Tanzania. Turkey, which is considered to fall between both Asia and Europe, contributes 261 isolates (estimated 5.29%). In comparison, Europe itself contributes very low numbers of 180 isolates (approximately 4.34%), mostly from Italy, with no records found in Germany, Norway, or Denmark. Finally, North and South America only contribute low numbers of STEC isolates, with almost no (0.14%) contributions from Canada and the United States, while South America contributes 343 isolates (approximately 8.27%), mainly from Brazil, and less from Chile and Peru (through oysters and fish farms) (Table 1).

#### 3.1.2. Aquaculture Sample Type

The STEC isolation rate by individual aquaculture sample type shows great disparity, indicating the presence of significant localized threats. In Asia, the maximum percentage of positive STEC samples is found in Malaysia, where oysters (Crassostrea iredalei) achieved the peak positivity of 86.67% (104/120) and downstream fishing villages 84.57% (148/175), followed by rainbow trout in Lebanon achieving 85.93% (116/135). India presents another example of high positivity in certain sectors, including shrimps at 77.45% (79/102) and pre-processing plants for seafood at 76.39% (110/144); however, the largest *Nile Tilapia* investigation in Thailand, which achieved only 19.08%, is considered moderate while having the largest number (828 isolates) among all studies. In Africa (Egypt), shrimp samples, finfish *(Nile Tilapia)* and *O. niloticus* cultivated in fish farms exhibit very high susceptibility, with 52.66% and 42.75% positivity, respectively. On the other hand, Bangladesh, South Korea, China (duck fish and grass carp), and India and Pakistan (fish, fish markets, clams, and shrimps) provide many sample types that have a negligible % of STEC isolates; thus, the STEC threat can be said to vary regionally and sector-specifically (Table 2).

### 3.2. Distribution of VAGs Among Aquaculture and the Associated Environment

Out of 4082 reported isolates from developing countries between 2015 and 2025, data on VAGs are available for 1419 cases (34.76%). Significant global heterogeneity indicates that certain aquaculture settings serve as critical reservoirs for highly virulent strains. In Africa, aquaculture environments exhibit significant heterogeneity, serving as critical reservoirs for highly virulent strains due to varying waste management and water sources. In Egypt, profiling reveals an unstable VAG distribution rate (4% to 54%) dominated by EHEC strains carrying classical Shiga toxins (*stx1*, *stx2*), intimin (*eae*A), and hemolytic proteins (*Omp*A, *hly*A) [20,21,22,23,24,25,26,27,28,29,30,31,32,33,34]. On the other hand, researchers who have done work in sub-Saharan Africa report a prevalence that is quite a bit lower. For example, river basins in Nigeria show an 11% distribution rate, mainly with *stx*2 and *hly*A as a characterizing feature [40], whereas Tanzanian groups have a very low 5% to 7% prevalence dominated almost entirely by the *stx*2 gene [44]. At the same time, country and ecosystem-specific virulence gene patterns show sharp differences in Asia’s aquaculture hotspots, which are also the area’s most densely packed with commercial activities. India has highly scattered contamination levels (0% to 100%) in coastal brines, freshwater ponds, and retail shops, with isolates generally carrying multi-virulent *stx*1, *stx*2, and *eae*A gene combinations [52,53,54,55,56,57,58,59,60,61,62,63,64,65,66,67,68,69,70,71,72,73,74]. Iran’s atypical typing levels are widely diverse (2% to 86%), acting as a special reservoir of certain allele versions such as *stx*2c and *stx*2d, as well as being a host for *eae*A and enterohemolysin *ehx*A [75,76]. In the same region, baseline monitoring figures differ greatly: Thailand ranges from 24% to 100% (*stx*1, *stx*2) [94,95,96], Malaysia reports an 86% level (*hyl*A) [85], and Pakistan shows a very localized 19% incidence featuring a typical *stx*1, *stx*2, and *eae*A profile [89]. However, thorough testing initiatives in China and South Korea have led only to baseline or unclassified results (Table S1).

Alternatively, European, North American (USA), and South American regions show a great difference in STEC levels, as Asian and Northeast African regions are hotspots while they are mostly below the detection level. Although regular monitoring in Germany, Switzerland, and some parts of Italy depicts a 0% baseline (or unclassified status), regional vulnerabilities still exist [109,111]. In fact, a 14% presence rate of *stx*1 and *stx*2 mixtures has been found in Italian shellfish and related environmental samples [110]. An equal 14% occurrence is found in Brazilian aquatic farming and fish systems, where molecular typing reveals a stable pathogen opportunity mainly characterized by the very toxic *stx*2 gene together with *ehx*A [121,122,124,125] (Table S1).

### 3.3. Shiga Toxins and Intimin Genes

Among 1419/1175 aquaculture isolates studied, *stx*1 and *stx*2 were the major virulence genes, whereas *eae* clusters were found in 827 isolates, which were mostly from wastewater sewerage systems. The VAG profiles differed when the data were broken down by commodity type. For example, out of 517 finfish isolates, 26.5% contained either *stx*1 or *stx*2, while 15.9% (*n* = 82) had *eae* co-expression—a profile mostly isolated from Iranian, Egyptian, and Indian retail markets [52,72]. Furthermore, the 406 shellfish and seafood isolates exhibited high pathogenic features; 22.4% contained *stx*1 or *stx*2, and 11.6% (*n* = 47) showed co-expression of multi-virulent *stx*1/*stx*2/*eae*. Interestingly, 28.6% of these shellfish isolates showed unique non-stx adherence profiles, mainly *hly*A or eae alone, which were mostly found in Malaysian and Brazilian production sectors (Table S1).

### 3.4. Detection of ARGs in Aquaculture and Associated Environments

Of the 4082 total cases recorded in fisheries and related habitats worldwide from 2015 to 2025, around 3055 (74.84%) ARGs were identified across different continents. In Northeast Africa, the intensive sectors of Egypt’s aquaculture contributed a major share of this resistance, with local strains of beta-lactamase and tetracycline resistance at high levels. In 90 cases, the fish species Nile Tilapia (Oreochromis niloticus) consisted of a complicated arrangement of genes for *tet*A and *Amp*C, and at the same time a separate group of 103 Tilapia isolates from earthen ponds revealed different ARG profiles with *bla*_CTX-M_, *bla*_CTX-M-15_, *bla*_SHV_, *bla*_AMP_, and *bla*_OXA-2_.

High gene density per positive sample was a signature characteristic of Asian aquaculture hubs. For instance, in India, two critical cases of specialized finfish and shellfish sectors displayed extreme multidrug resistance (MDR) capacity, resulting in 52 unique ARGs only in two isolates [52]. These isolates contain sophisticated resistance mechanisms like the carbapenemase gene *bla*_NDM_ and *tet*G, *tet*O, *tet*W, *qnr*A, and *qnr*B. Similarly, the monitoring of Pakistan’s carp fish farming and surface water and sediments found a combined total of 79 ARG detections, dominated by *bla*_CTX-M_ and *bla*_TEM_ and also containing *tet*A, *sul*1, *sul*2, *qnr*A, and *qnr*B [87,88,89,90]. In a similar way, Thailand’s coastal aquaculture environments in the SEA region displayed a heavy resistance footprint; 67 positive cases in oysters and estuarine waters accounted for a shockingly high 158 total ARG detections, almost entirely driven by *bla*_TEM_, *bla*_CTX-M_, and *tet*A combinations [94,95,96,97] (Table S2).

In the Middle East, fish sold in the Iranian retail sector displayed 127 structural resistance markers over 49 cases, which reveals a highly diverse profiling mix of *tet*A, *tet*M, *sul*1, *sul*2, *qnr*A, *qnr*B, *dfr*A14, and *bla*_OXA_ [75,78,79]. In the Americas, Brazil showcased a well-established MDR scheme where 65 total cases covering fish, shrimp, oysters, and aquaculture water revealed 57 certain ARGs including *bla*_TEM_, *bla*_CTX-M_, *bla*_CMY-2_, *qnr*A, *qnr*B, and *sul*1. Interestingly, a particular group of 23 oyster samples featured clear distributions of *bla*_TEM_, *bla*_SHV_, and the very important carbapenem resistance marker *bla*_KPC_ [118,120,125]. Then again, routine monitoring in Bangladesh, South Korea, Germany, Denmark, Kosovo, Chile, and Peru did not find any relevant ARG presence in aquaculture systems or their surrounding environments (Table S2).

### 3.5. Serogroups

Across 3052 VAG-based isolates, 1118 (36.63%) aquaculture samples were heavily dominated by serogroups O157, O26, and O111. In 1577 cases (95.4%), O157:H7 was tracked in 25 cases (1.5%) [20,26,36,37,38,87]. Serological testing on 547 finfish isolated different serogroups; O157, O26, and O111 dominated a total of 291 cases (53.2%) [80,86]. The individual high-risk serotypes O157:H7 and O118 (or O111:H8 variants) made up 44 (8.0%) and 90 (16.5%) cases, respectively [27,35]. In contrast, among 464 shellfish and seafood isolates, serogroups O157, O26, and O111 were found in 242 cases (52.2%). Interestingly, the dangerous O157:H7 serotype was more common in the shellfish group, making up 110 cases (23.7%), whereas the specialized O113:H21 serotype came next in 65 cases (14.0%) (Table S2).

## 4. Discussion

A worldwide meta-analysis of combined data shows that even though STEC has extensively spread over aquaculture systems and related environments, its prevalence is still very uneven. The isolation of STEC from the world’s major aquaculture producers, like Malaysia, India, Brazil, and Egypt, highlights the cross-continental aspect of this biosecurity threat [128]. An important result from this data is the big difference between the total number of samples and the number of positive samples obtained. This massive difference between the sampling denominator and the positive numerator proves that STEC contamination is not evenly spread but is found in clusters, which points to particular ecological niches that can be targeted for regulatory intervention.

One of the main factors determining the environmental persistence of STEC in aquaculture systems is its capacity to colonize different types of substrates. The pathogen’s higher recovery rates in benthic sediments, processing machinery, and effluent runoff versus open water bodies imply that stagnant hydraulic conditions and organic enrichment enhance the survival of these enteric organisms [129]. In fact, sediments are not only a part of the environment; on the contrary, they offer bacterial populations protection from solar ultraviolet radiation and predatory microbes, leading to their long-term survival and to their resuspension into the water column during heavy rains or harvesting operations. Beyond substrate colonization, STEC spread in aquaculture is driven by favorable fluctuations in water temperature and pH, reduced competition from native microbial communities, and the formation of sanitizer-resistant biofilms on processing equipment [17]. However, the health and animal threat that aquaculture-related STEC may cause is mainly regulated by the detailed characterization of its toxins. The gathered data indicate a very sparse distribution of the main pathogenicity factors, i.e., the Shiga toxin variants (*stx*1 and *stx*2) and *eae* (Table 3).

The relative proportions of *stx*1 to *stx*2 locally exhibited marked geographical differences among the isolates examined. So, *stx*1 was the most common toxin type in several African and Asian aquaculture-related datasets, whereas in the European and South American ones, *stx*2 showed either a higher or a similar prevalence as stx1. This probably occurred because integrated farming practices introduce animal manure and agricultural runoff into aquaculture environments. As a result, *E. coli* strains in these waterways reflect local animal shedding, with small ruminants and wildlife driving a high prevalence of stx1-carrying strains in the water, sediment, and seafood. From an epidemiological viewpoint, this difference is quite important; human STEC infections with strains harboring *stx*2 are generally connected to very severe clinical symptoms such as hemorrhagic colitis (HC) and even life-threatening HUS; in contrast, those strains carrying *stx*1 usually cause self-limited, mild diarrhea [130]. So, the identification of *stx*2-positive strains in commercial food-producing aquatic environments points to a significant zoonotic threat for consumers. The *stx*2 gene plays a crucial role in zoonotic outbreaks because it encodes Shiga toxin type 2, which has a significantly higher binding affinity for human endothelial receptors than *stx*1. This allows the toxin to effectively halt host protein synthesis, causing severe clinical complications such as HC and life-threatening HUS. This is because it has an exceptionally low infectious dose and is easily shed from animal reservoirs into food chains [128]. In 2019, a multi-state US outbreak linked to raw oysters imported from Mexico resulted in 16 confirmed illnesses, with non-O157 STEC identified as a critical co-infecting pathogen alongside *Vibrio* and *Shigella* species [116].

The presence of *eae* provides valuable context regarding how these environmental isolates colonize hosts. Strains carrying the *eae* gene represent typical Shiga toxin-producing STEC pathotypes, which can produce characteristic attaching-and-effacing (A/E) histopathological lesions on intestinal epithelial tissue [131]. However, a notable proportion of the strains evaluated in this literature review harbored core *stx* genes but lacked the *eae* marker. These atypical STEC variants are common in environmental samples. Their presence in aquaculture systems points to two likely scenarios: either these strains utilize alternative chromosomal or plasmid-encoded adhesins such as *saa* or *iha* to adhere to cells, or these production systems are experiencing temporary biological inputs from agricultural or wildlife runoff, where *eae*-negative populations are widespread. Beyond these virulence profiles, a highly concerning finding from our meta-analysis is the widespread co-carriage of ARGs within these environmental STEC populations. This evidence strongly indicates that aquaculture infrastructure acts as an active, critical reservoir that preserves and facilitates the horizontal gene transfer of AMR [132].

Among the ARGs found in the literature, genes that provide resistance to old antimicrobials predominantly consist of tetracyclines (*tet*A, *tet*B), β-lactams (*bla*_TEM_), and sulfonamides (*sul*1, *sul*2), which showed a high level of baseline presence. These highly prevalent resistances represent the effects of prolonged and repeated selective pressures due to the historical overuse or misuse of these drug classes as prophylactics or growth promoters, not just in aquatic environments under intensive production but also in terrestrial animal husbandry [131]. Yet, what is more alarming from a clinical perspective is the rise in the detection of genes that confer resistance against major human drugs, like the genes for ESBLs (*bla*_CTX-M_), those that give resistance to quinolones in plasmid form (*qnr*S), and even *mcr*-1. Furthermore, driven by veterinary interventions and heavy metal runoff, aquaculture environments exert continuous co-selection pressure that actively selects for bacteria carrying critical ARGs. Concurrently, the protective biofilms formed by STEC on pond sediments and processing machinery trap these microorganisms in close proximity. This localized, high-density environment accelerates HGT, allowing native aquatic bacteria to readily transfer mobile plasmids carrying last-resort resistance genes like *bla*NDM and *mcr*-1 directly to STEC [89]. The finding of very dangerous resistance genes like *bla*_NDM_ or *mcr*-1 that compromise the main food production chains is a catastrophic event for the modern clinical medicine, as it could render ineffective the drug combinations that are vital in human medicine for the treatment of the most serious system-wide infections [133].

The VAGs and ARGs are found together in single bacteria, highlighting the huge evolutionary benefits of these environmental pathogens. In water bodies, long-term exposure to very low levels of stress factors such as leftover antibiotics, heavy metals, and disinfectant residues continuously pushes organisms to develop MDR [133]. Since many ARGs (*bla*_TEM_, *tet*, *sul*, *qnr*S) and certain atypical virulence characters are located on the same mobile genetic elements (MGEs) like conjugative plasmids, transposons, and class 1 integrons, giving selective pressure by only one antimicrobial agent might cause the simultaneous co-selection and stabilization of the entire virulence-resistance genetic cassette. Sediments and microbial biofilms at the bottom of the fish-farming ponds act as high-density cellular matrices, which greatly enhance the rate of horizontal gene transfer (HGT) [131]. So, harmless commensal *E. coli* or other aquatic microbiota can easily take in these mobile genes; that is, it turns the aquaculture ecosystem into a genetic chamber from which new, hyper-virulent, and pan-resistant pathotypes constantly evolve [134].

Monitoring STEC and its related VAGs and ARGs in aquaculture systems has revealed external anthropogenic and agricultural inputs as the main culprits for environmental contamination. Since *E. coli* is an enteric organism and not a natural aquatic pathogen, its presence in production ponds, water inflows, and farm equipment is a clear indication of fecal contamination [133]. Municipal wastewater discharges, untreated agricultural runoff from adjacent livestock operations, and the conventional use of raw animal manure for pond fertilization (integrated livestock–fish aquaculture) are the main transmission vehicles [135]. After introduction into the environment, the pathogen shows remarkable ecological plasticity, allowing it to efficiently adapt to the aquatic habitat. The evidence lies in the collected data that demonstrate recovery of the pathogen not only within the primary water column but also on processing equipment, workers’ hands, and internal anatomical sites (including the abdominal cavity and branchial filaments) of cultured fish species. Such widespread presence along the post-harvest supply chain indicates that the aquatic environment is more than just a passive vehicle for the transport of pathogens. It is, in fact, an active matrix where pathogens survive, build up, and horizontally exchange resistance traits [136].

The rise in toxigenic STEC strains with crucial virulence factors and complicated antimicrobial resistance profiles in aquatic farming environments is posing a complex risk to global public health. The contamination of seafood, mainly when the fish is caught from highly polluted areas with sediment or water, is a direct yet frequently overlooked route to transmission. The isolation of MDR-STEC from the hands of workers and processing tools at fish farms indicates a direct pathway for local cross-contamination within the post-harvest supply chain. Most importantly, therapeutic choices would be very limited for treating clinically infected individuals in foodborne outbreaks attributed to these pathotypes due to their acquired ARG profiles [137].

Though the present paper describes a decade-long overview, some limitations still need to be studied in future like the literature’s bias towards commercial aquaculture and standard culturing techniques, which fails to take into account unculturable STEC, thus distorting the actual prevalence of genes. Additionally, the lack of longitudinal studies in original scientific papers makes it difficult to distinguish between temporary runoff from terrestrial sites and permanent ARG integration into the aquatic microbiome. Moreover, current studies are biased towards basic molecular methods of identifying ARGs and VAGs without being able to prove their co-localization on mobile genetic elements, i.e., plasmids, transposons, etc. These outcomes highlight the necessity for an integrated “One Health” approach that aligns regular molecular monitoring of VAGs and ARGs with strict cleaning requirements and upstream fecal management methods to maintain both food safety and clinical effectiveness.

## Figures and Tables

**Table 1:** Total distribution of STEC isolated from different aquaculture sources.

Continent		Total No. of Isolates		STEC Isolates	VAGs	ARGs
Africa		5655		1009	270	657
Asia		8437		2283	926	1938
Asia/Europe		1579		261	7	124
Europe		1714		180	93	184
North America		375		6	ND	55
South America		5426		343	123	97
		23,186		4082	1419	3055
ANOVA						
Source	SS	Df	MS	F	*p*-value	F crit
Between Groups	“52,285,211”	3	“17,428,403.6”	6.273698673	0.003551 *	3.098391
Within Groups	“55,560,219”	20	“2,778,010.95”			

* alpha = 0.05; ND = not detected.

**Table 2:** Within-continent prevalence of various VAGs and ARGs in Shiga toxin-producing *E. coli* from aquaculture and its associated environments.

Continent	Country	Source	Total No. of Isolates	STEC Isolates	VAGs	ARGs	Reference
Africa	Egypt	Fish farm water (fish, *O. niloticus*, *S. pilchardus*	328	76	*stx*1, *stx*2, *eae*A, st, lt, bfpA	*bla*TEM, *bla*CTX-M, *blaOXA*-48	[20]
		*Clarias gariepinus* catfish	50	10	*stx*1, *stx*2,	ND	[21]
		*Oysters*	33	27	*sfa*, *exh*A, *eae*A, *est*A, *aer*, *stx*1, *stx*2, *rfc*, *hly*A, *etr*A, *cnf*1, *sep*A,	*bla*TEM, *bla*SHV, *bla*CTX−M, *bla*OXA−1	[22]
		Finfish (Tilapia, mullet)	150	79	*stx*1, *stx*2	MDR gene	[23]
		Mullet meat	100	20	SLT, LT	*aaC*2 gene	[24]
		Gandolfi, *Ruditapes decussatus*	20	20	*stx*1, phoA, stx2	ND	[25]
		Shrimp (*L. vannamei*)	82	11	ND	*bla*TEM, *tet*A, *bla*OXA, *sul*1, a*ad*A, *erm*B	[26]
		Seafood	135	57	*stx*1, *stx*2, *eae*A,	ND	[27]
		Seafood samples	192	10	*stx*1, *stx*2 and *eae*A	*bla*TEM, *tet*A	[28]
		Migratory birds	349	4	*stx*1, *stx*2 and *eae*A	MDR gene	[29]
		Fish	150	10	*stx*1, *stx*2, and *eae*A	ND	[30]
		Fish samples from gills, musculature	200	9	*stx*1, *stx*2 and *eae*A	ND	[31]
		Earthen pond-cultured *O. niloticus*	144	11	ND	*bla*TEM, *bla*CTX-M, *bla*SHV, *bla*AMP *bla*OXA-2	[32]
		Tilapia (*O. niloticus*), *Clarias gariepine*	400	171	*stx*1, *stx*2 and *eae*A	*tet*A, *Amp*C	[33]
		Fresh Nile Tilapia fish	218	24	*stx*2, *eae*A	*tet*A, *bla*TEM, *sul*1, *Sul* 2, *aad*A2	[34]
	Ethiopia	Fish	450	8	*stx*1, *stx*2, *eae*A,	*bla*TEM-1B, *tet*A, *fos*7, *qnr*S, *gy*rA	[35]
		Fish	410	1	*stx*1, *stx*2	ND	[36]
		Fish	121	41	*eae*, *hly*A1		[37]
		Fish	328	62	Nil	ND	[38]
	Ivory Coast	Fish, crab, water sediment	62	9	*stx*1, *stx*2, *eae*, *Agg*R	elt, est	[39]
	Nigeria	Miscellaneous retail fish	256	28	ND	ND	[40]
		Fish, shellfish	72	11	*stx*2, *hly*A	*bla*SHV, *bla*TEM, *mcr* genes	[41]
		Nile Tilapia, fish farms	50	-	ND	ND	[42]
		Clarias gariepinus (catfish and aquaculture)	47	5	ND	ND	[43]
	Tanzania	Manure, vegetables, and fish	36	2	*Stx*2	ND	[44]
	West Africa	Fish farm	156	99	ND	ND	[45]
	Kenya	Nile Tilapia fish	68	24	ND	*tet*A, *bla*TEM, *sul*1, *Sul* 2, *aad*A2	[46]
	Zambia	Fish	418	56	*stx*1, *stx*2 and *eae*A	*bla*SHV, *bla*TEM, *bla*CTX-M	[47]
	Zimbabwe	Water sample (natural lake)	600	120	ND	ND	[48]
		Fish	30	4	ND	ND	[49]
Asia	Bangladesh	Fish	312	139	ND	ND	[50]
		Shrimps (water, basket, mat)	58	29	ND	ND	[51]
	India	Shrimp farms (pond water)	102	79	*stx*2	*bla*TEM, *bla*CTX-M, *bla*SHV, *Amp*C, *sul*1	[52]
		Fish (freshwater pond sites, retail)	243	18	ND	*bla*TEM, *bla*CTX-M-15, *bla*OXA-1, *tet*A, *sul*1, *qnr*S, *cat*1, *cat*2	[53]
		Seafood samples	63	34	ND	ND	[54]
		Seafood samples	130	60	ND	*bla*CTX-M, *bla*SHV, *bla*TEM, AmpC	[55]
		Carp, aquaculture	232	49	*stx*1, *stx*2	MDR gene, enteric genes	[56]
		Seafood pre-processing plants	144	110	*stx*1, *stx*2, *eae*A	*uid* A gene	[57]
		Seafood	43	21	ND	*Rfb*	[58]
		Fish supply chain	91	21	ND	*Sul*1, *bla*CTX-M, *tet*D, *dfr*A, *Amp*C	[59]
		Fish	70	28	ND	ND	[60]
		Shrimps	144	18	*stx*1, *stx*2, *eaeA*, *hly*A	*uid* A gene	[61]
		Salmon sashimi (fish)	30	15	ND	ND	[62]
		Food fish	79	5	ND	*bla*TEM, *bla*SHV, *blaOXA*, *tet*A, *Str*A, *Sul*1, *Sul*2, oqxA, *qnr*B	[63]
		Fish	100	6	ND	ND	[64]
		Finfish, shellfish	134	2	*pap*AH, *pap*C, *sfa*/focDE, *iut*A, *kps*MT-II	*bla*TEM, *bla*SHV, *bla*CTXM, *bla*NDM, *tet*A, *tet*B, *aph*A2, *st*rA, *sul*1, *sul*2, dhfrIa	[65]
		Fish	200	76	ND	*bla*TEM, *bla*SHV, *bla*CTX-M	[66]
		Carp from integrated aquaculture ponds	30	1	*stx*1, *stx*2, *eae*, *ehly*A, *rfb*O157	Rfb	[67]
		Fresh seafood	100	9	*stx*1d, *stx*2, *eae*A, *hly*A	ND	[68]
		Carps	33	9	*stx*1, *stx*2, *eae*, *ehly*A, *rfb*O157	ND	[69]
		Fish market waste	35	1	ND	ND	[70]
		Fish	238	28	ND	ND	[71]
		Freshwater fish (*Catla catla*)	104	18	*stx*1, *stx*2, *eaeA*, *hly*A	ND	[72]
		Clams and shrimps	160	12	ND	*bla*VIM	[73]
		Shrimp farms	261	9	viz., *iut*A, *ent*B, *mr*kD	*bla*TEM, *tet*A, *tet*B, *Sul*1, *Su*l2, *cat*A, *Cml*A, *qnr*S	[74]
	Iran	Fish (retail store)	166	65	*stx*1a, *stx*1c, *stx*1d, *stx*2c, *stx*2d, *stx*2e *stx*2f	*bla*TEM, *qnr*A, *tet*B, *sul*2, *blaSHV*, *sul*, *blaCTX*-M, *bla*OXA	[75]
		River and sewage water	70	14	*stx*1, *eae*	ND	[76]
		Cold-smoked salted fish	200	5	ND	ND	[77]
		Aquaculture water	150	27	ND	*tet*A, *Cml*a, acc-IV, *qnr*A, *sul*1, *sul*2	[78]
		Cold water fish	100	14	*stx*1, *stx*2, *eae*	MDR genes	[79]
	Iraq	Cultured fish (*Labeo rohita*, *Cyprinus*)	120	11	ND	*bla*TEM, *bla*CTX-M	[80]
		Water, sediment, feed, tools	230	40	ND	ESBL producing isolates	[81]
		Fish farms, fish markets	150	40	ND	MDR gene	[82]
	Jordan	Recreational lake water	262	130	ND	MDR genes	[83]
	Lebanon	Fish, rainbow trout, aquaculture	135	106	ND	*mcr*-1	[84]
	Malaysia	Oyster (*Crassostrea iredalei*)	120	104	*Hly*A	ND	[85]
		Downstream of a fishing village	175	148	ND	MDR gene	[86]
	Pakistan	Freshwater fish-farming sites	101	55	ND	*qnr*A, *qnr*B, *qnr*S	[87]
		Fish (*Channa marulius*)	480	29	ND	*tet*A, *sul*3, *gyr*B, *bla*TEM	[88]
		Fish, shrimp, aquaculture water	225	65	*stx*1, *stx*2, *eaeA*	*bla*NDM-1, *mcr*-1	[89]
		Cerus (*Labeo rohita*, *Catla catla*)	216	25	ND	*bla*SHV, *bla*TEM, *bla*CTX-M, *tet*A, *tet*B, *erm*A, *erm*B, *erm*C, *vga*A, *str*A, *str*B, *aad*A1, *aac*3-I	[90]
	Singapore	Aquaculture farms	31	23	*stx*, intL	*bla*SHV, *bla*OXA, *bla*CTX-M, *qnr*A	[91]
	South Korea	Coastal aquaculture	22	22	ND	*str*A, *str*B, *aad*A1, *aac*3-I	[92]
		Fish (gizzard shad, halibut, rockfish)	206	49	ND	ND	[93]
	Thailand	Nile Tilapia	828	158	*stx*, *eae*A,	*bla*TEM, *tet*A, *qnr*S	[94]
		Oysters and estuarine water	409	8	*stx*1, *stx*2	*bla*TEM, *te*tA	[95]
		Oysters, estuarine water	109	59	*stx*1, *stx*2, *eae*	*bla*TEM, *tet* A, *bla*SHV, *stn*, *sul*2	[96]
		Aquatic environment, coastal waters	384	84	ND	*bla*TEM, *blaCTX*-M-15, *blaOXA*-1, *tet*A, *sul*1, *qnrS*	[97]
		Fish	42	22	*scnf*2s, LTIs or vt2e	ND	[98]
	China	Fish (carp)	20	8	ND	ND	[99]
		Aquaculture farms, soil sediment	90	28	ND	*bla*TEM, *bla*NDM, *flo*R, *Optr*A, *cml*A, *aph*A1, *Sul*2, *oqx*A, *qn*rS	[100]
		Duck fish	250	143	ND	*mcr*-1	[101]
		Grass carp fish	10	9	ND	*bla*CTX-M 27, *bla*CTX-M15	[102]
Asia/Europe	Türkiye	Fish (*Engraulis encrasicolus*, *Merlangu*)	300	157	*stx*1, *stx*2	ND	[103]
		Raw mussels	71	2		*Sul*3, *tet*B, *qnr*A, *qnr*B, *sul*1, *fimH*, *flo*R	[104]
		Fish	140	2	*stx*1, *stx*2	ND	[105]
		Mussels, sea bass	823	100	ND	*bla*TEM, *tet*A	[106]
		Fish	245	-	ND	ND	[107]
Europe	Denmark	Zebra fish	300	67	ND	*bla*TEM, *Su*l2, *qnr*S, *mcr*-1	[108]
	Germany	Fish in Baltic sea	116	41	ND	ND	[109]
	Italy	Clams and shrimps	620	14	*stx*1, *stx*2, *eaeA*, *hly*A	*bla*VIM, *acc*, *bla*SHV	[110]
		Carpet shells (*Cerastoderma* spp.)	40	20	*stx*1, *stx*2	ND	[111]
	Kosovo	Salmon fish	54	2	ND	ND	[112]
	Norway	Marine bivalve mollusks	540	10	ND	*bla*CTX-M	[113]
	Switzerland	Shellfish (oysters, shrimps)	44	26	ND	ND	[114]
North America	Canada	Seafood	277	2	ND	*bla*KPC, *bla*OXA, *bla*NDM	[115]
	USA	Seawater, shellfish (oysters)	8	4	ND	ND	[116]
		Nile Tilapia	90	-	ND	*bla*TEM, *blaCTX*-M, *bla*SHV, *amp*C, *sul*1	[117]
South America	Brazil	Fish, shrimp, oysters, aquaculture water	225	65	ND	*bla*TEM, *bla*CTX-M, *bla*CMY-2, *tet*A, *tet*B, *sul*1, *sul*2, *qnr*A, *qnr*B, *qnr*S, *aad*A, *flo*R, *mcr*-1	[118]
		Culture farms (fish, shrimp, oysters)	69	29	ND	ND	[119]
		Aquaculture farms (fish, shrimp, oysters, seafood)	6	5	ND	*bla*TEM, *bla*CTX-M, *bla*CMY-2, *tet*A, *sul*1, *qnr*S	[120]
		Mussels	21	21	*stx*1, elT, bfpA	ND	[121]
		Shellfish	150	1	*pho*A, *eae*	ND	[122]
		Frozen Tilapia filets (processing units)	100	50	ND	ND	[123]
		Fish, fish ponds	115	13	*eae*A, *stx*1, *stx*2	ND	[124]
		Oysters	940	23	*eae*A, *stx*1, *stx*2, Ial, *ST*a, *ST*b, LT	*bla*TEM, *bla*SHV, *bla*KPC	[125]
	Chile	Fresh seafood	330	18	*hyl*A, *stx*2, *ipf*A	*pmr*B, *qnr*B	[126]
	Peru	Fish	500	118	ND	ND	[127]

ND = not detected.

**Table 3:** Global overview of microbial contamination, aquaculture pathogens, and detection methodologies across sampled continents.

Continent	No. of Studies	Representative Sources Sampled	Human Risk Categories Detected	Animal Fecal/Tissue Contamination Markers	Primary Aquaculture Pathogens (Strains)	Methodologies & Techniques Employed
Asia	53	Cultured fish Labeo rohita, Cyprinus carpio, fish, freshwater fish-farming sites	Potential public health risk, hand swabs	Fecal contamination, skin, gills, intestines	STEC, *E. coli* O157:H7, STEC, EPEC	PCR, culture, genotyping, MALDI-TOF
Africa	30	Nile Tilapia O. niloticus, shrimp L. vannamei, dish farm water	N/A, zoonotic risk identified	Yes—92.68%, tissue	*E. coli* O157:H7, STEC O157, STEC	Culture, 16S rRNA PCR, PCR, pathogenicity trial
Europe	12	Fish Engraulis encrasicolus, Merlangius euxmus, Trachurus mediterraneus, Mullus barbutus, Tinca tinca, Cyprinus carpio and Oncorchynhus mykiss, raw mussels, fish	N/A	Beef, meat	*E. coli* O157:H7, *E. coli*, STEC, APEC	mPCR, E-test method, Electrophoresis, RT-PCR
South America	10	Fish, shrimp, oysters, aquaculture water, aquaculture farm fish, shrimp, oysters, aquaculture pond water, seafood, meat, vegetables and ready-to-eat street-vended food	Yes	Fecal contamination	STEC, EPEC, ETEC, STEC, *E. coli* O157:H7, STEC	PCR, AMR profiling, RT-PCR, qPCR
North America	3	Seawater, shellfish, oysters, seafood, fish feces	Fecal contamination	Fecal contamination	STEC O157, *E. coli*, CRE, *E. coli* O157:H7	PCR, qPCR, Multiplex PCR
Total Studies	108					

## Data Availability

No new data were created or analyzed in this study. Data sharing is not applicable to this article.

## Supplementary Materials

The following supporting information can be downloaded at: https://www.mdpi.com/article/10.3390/vetsci13080779/s1, File S1: PRISMA 2020 checklist [138]; Table S1: Distribution of VAGs in Shiga-toxin producing *E. coli* from Aquaculture and its associated Environment; Table S2: Continent wise distribution of ARGs in STEC from Aquaculture and its associated Environments.

## Abbreviations

The following abbreviations are used in this manuscript:

STEC	Shiga toxin-producing *E. coli*
ARGs	Antibiotic resistance genes
VAGs	Virulence-associated genes
MDR	Multidrug Resistance
HGT	Horizontal gene transfer
AMR	Antimicrobial resistance
EPS	Extracellular polymeric substance
HUS	Hemolytic uremic syndrome
